# Supplementary material for: The Effect of Functionalized Multiwall Carbon Nanotubes with Fe and Mn Oxides on Lactuca sativa L
Source: Plants (Basel). 2023 May 11;12(10):1959. doi: 10.3390/plants12101959 (PMC10222390; doi:10.3390/plants12101959)
Supplement: Supplementary file 1 [file plants-12-01959-s001.zip › plants-2348820-supplementary.pdf]

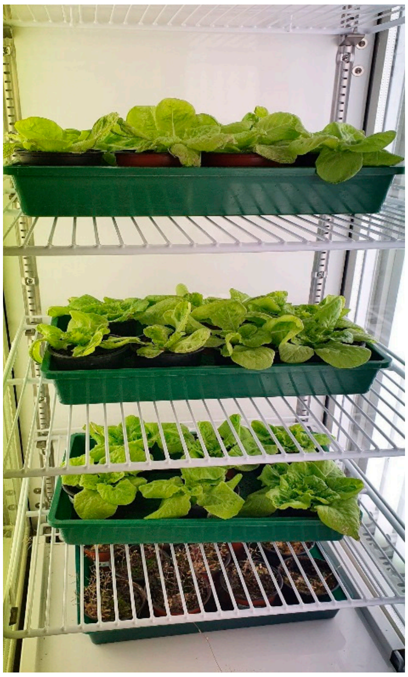

**Figure S1.** Plant development at 21 days.

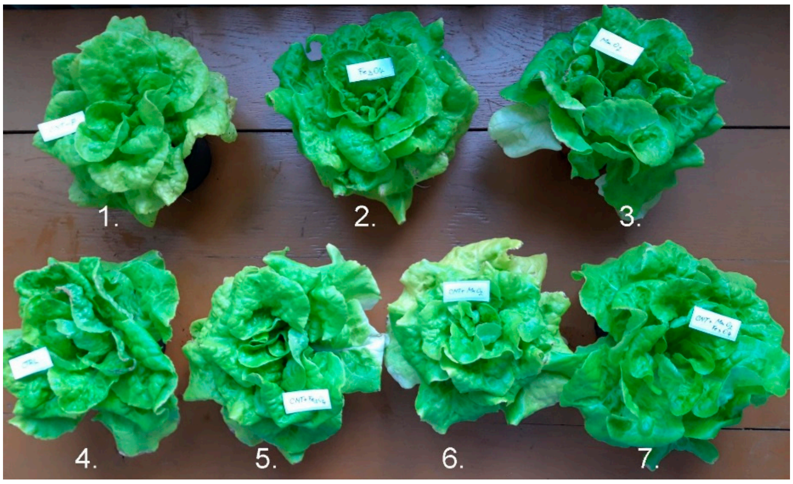


**Figure S2.** One individual plant from each treatment: 1- CNT-COOH; 2- $Fe_3O_4$; 3- $MnO_2$; 4- Control; 5- CNT-$Fe_3O_4$; 6- CNT-$MnO_2$; 7- CNT-$Fe_3O_4$-$MnO_2$.

**Table S1.** Elemental composition of lettuce leaves described in the literature.

| | Na* | Mg* | Al** | Cl* | K* | Ca* | Mn** | Sc*** | Fe** | Co*** | References |
|---|---|---|---|---|---|---|---|---|---|---|---|
| I[a]; m.a. | 0.14±0.03 | | | | 6.3±1.4 | 0.51±0.08 | | | 16.1±3.0 | 7.9±1.7 | [44] |
| O[a]; m.a. | 0.34±0.07 | | | | 8.3±1.9 | 1.17±0.18 | | | 44±13 | 19.3±5.6 | |
| I[b] ; m.a. | 0.17±0.14 | | | | 7.2±0.8 | 0.54±0.05 | | | 16.9±4.6 | 8.4±1.7 | |
| O[b] ; m.a. | 0.31±0.11 | | | | 10.7±1.7 | 1.29±0.06 | | | 53.2±3.6 | 30±10 | |
| | | | | | | | | | 530.4[1] | 7200[1] | [40] |
| m.a. | 2.9±0.7 | 3.6±0.7 | 3524±318 | 17.9±0.4 | | 13.3±0.6 | 68.81±0.22 | | 1506±54.3[2] | 1010±160[2] | |
| C ; c.s. | 4.2 | 5.2 | | 22.8 | 96.1 | 30.3 | 0.042 | | 0.42 | 302 | [35] |
| c.s. | 6-10.3 | 3.6-6.8 | | 8.5-15.1 | 52.7-74.9 | 14.9-22.9 | 0.013-0.038 | | 0.19-0.97 | 76-1026 | |
| O; m.a. [3] | 1.93±0.01 | 3.2±0.014 | | | 84.8±1.7 | 12.9±0.15 | 157.6±3.1 | | 343±24 | 0.13±0.05 | [37] |
| O; m.a.; [2] | 2.4±0.8 | | | | 82±13 | 9.92±0.45 | | | 409±28 | 0.23±0.08 | |
| I; m.a.; [3] | 0.74±0.04 | 2.04±0.01 | | | 54.3±0.1 | 5.0±0.28 | 38.2±1.2 | | 102±10 | 0.067±0.02 | |
| I; m.a.;[2] | 1.3±1.1 | | | | 55.1±6 | 4.18±0.04 | | | 130±35 | | |
| c.s. | | 3.9-6.1 | | | 35.7-56.0 | 16.7-26.3 | 43.9-89.2 | | 160-514 | | [36] |
| C | | 5.1 | | | 66.0 | 19.0 | 136 | | 316 | | |
| c.s. | 1.3-9.1 | 3.8-4.5 | 5.6-103 | | 12.8-27.3 | 3.8-14.6 | 8.0-41 | | 41-159 | 30-190 | [39] |
| | | | 73 | | | | | 7-12 | | | [34] |
| p.w.c. | | | 1634- 6450 | | | | 113-170 | 70-37100 | 349-1087 | 0.2-72 | [46] |
| u.c. | 1.2-3.7 | 2.3-4.2 | | 54-125 | 36-93 | 4.3-7.6 | 10-23 | | 91-393 | 1000-21000 | [38] |

**Table S1.** Continuation.

| | **Zn**** | **As***** | **Se***** | **Br**** | **Rb**** | **Sr**** | **Mo**** | **Sb***** | **Cs***** | **Ba**** | **Th***** | **References** |
|---|---|---|---|---|---|---|---|---|---|---|---|---|
| I[a]; m.a. | 4.99±0.58 | 13±6.7 | | 1.27±0.23 | 1.19±0.1 | 2.45±0.39 | | <8 | | | | |
| O[a]; m.a. | 3.55±0.60 | 43±16 | | 2.74±0.45 | 1.56±0.11 | 5.5±0.54 | | 3.94±0.86 | | | | [44] |
| I[b] ; m.a. | 7.24±0.5 | 6.5±2.1 | | 3.41±0.45 | 1.20±0.12 | 2.00±0.23 | | 4.5±1.5 | | | | |
| O[b] ; m.a. | 7.14±0.33 | 24.3±2.1 | | 8.3±1.8 | 1.72±0.18 | 6.85±0.59 | | 11.5±1.8 | | | | |
| m.a. | 67.8[1] | | | | | | | | | | | [40] |
| C ; c.s. | 0.84 | | | 87.5 | 52.3 | | | 85 | 50 | | 25 | [35] |
| | 0.35-1.23 | | | 62-80 | 59.3-81 | | | 30.3-79 | 170-2360 | | 24-143 | |
| O; m.a. [3] | 45.7 | | | | 11.6±0.94 | | | 46.8±7.1 | | | | |
| O; m.a.; [2] | 54.9 | | | | 13.2±1.4 | | | 88±14 | | | | [37] |
| I; m.a.; [3] | 56.2 | | | | 9.22±0.34 | | | 34.9±7.3 | | | | |
| I; m.a.;[2] | 55.7 | | | | 9.23±0.92 | | | 35±12 | | | | |
| c.s. | c.s. | 70.5-439 | | | | | | | | | | [36] |
| C | C | 83.0 | | | | | | | | | | |
| c.s. | c.s. | 28.7-37.5 | 310-490 | | | | 22.7-34.9 | 0.27-0.36 | | | 5.2-12.0 | [39] |
| | | 55-530 | 20-250 | 57 | 20-22 | 14 | 74 | 0.074 | | | 9.4 | [34] |
| p.w.c. | 25-42 | | | | 13-57 | | | 100-4200 | | 10-135 | 150-20000 | [46] |
| u.c. | 56-52 | | | 17-32 | 68-142 | | | | 160-450 | 7-27 | | [38] |

*g/kg; **mg/kg; *** μg/kg;

C- control lettuce leaves; O-outer lettuce leaves; I-inner lettuce leaves; [a]-Evora; [b]-Coimbra; [1]-AAS, [2]-INAA; [3]-k0-NAA

m.a. - market available; c.s. – contaminated soil; p.w.c.- potential polluted water for irrigation; u.c –urban cultivation
